# PPAR*γ* Alleviates Sepsis-Induced Liver Injury by Inhibiting Hepatocyte Pyroptosis via Inhibition of the ROS/TXNIP/NLRP3 Signaling Pathway

**DOI:** 10.1155/2022/1269747

**Published:** 2022-01-30

**Authors:** Zeyu Li, Tong Liu, Yang Feng, Yingmu Tong, Yifan Jia, Cong Wang, Ruixia Cui, Kai Qu, Chang Liu, Jingyao Zhang

**Affiliations:** ^1^Department of Hepatobiliary Surgery, The First Affiliated Hospital of Xi'an Jiaotong University, Xi'an, Shaanxi 710061, China; ^2^Department of General Surgery, Shaanxi Provincial People's Hospital, Xi'an, Shaanxi 710068, China; ^3^Department of Rehabilitation Medicine, The Affiliated Hospital of Northwest University, Xi'an No. 3 Hospital, Xi'an, Shaanxi 710021, China; ^4^Department of SICU, The First Affiliated Hospital of Xi'an Jiaotong University, Xi'an, Shaanxi 710061, China

## Abstract

Sepsis is a systemic inflammatory response syndrome caused by a dysregulated host response to infection. Peroxisome proliferator-activated receptor gamma (PPAR*γ*) exerts anti-inflammatory and antioxidative properties. To investigate the potential effects of PPAR*γ* on sepsis-induced liver injury and determine the related mechanisms, C57BL/6 male mice were subjected to cecal ligation and puncture (CLP) to create a sepsis model which was treated with GW1929 or GW9662 to upregulate or downregulate the expression of PPAR*γ*. We found that upregulation of PPAR*γ* decreased the serum aspartate aminotransferase (AST), alanine aminotransferase (ALT), total bilirubin (TBIL), and liver pathological damage and improved the 5-day survival rate. Increased expression of PPAR*γ* also decreased sepsis-induced reactive oxygen species (ROS) by promoting the expression of Nrf2. In addition, upregulated PPAR*γ* inhibited the expression of the TXNIP/NLRP3 signaling pathway by reducing ROS-induced injury in the liver during sepsis, which further reduced NLRP3-mediated pyroptosis and the inflammatory response. The role of PPAR*γ* was further examined in in vitro experiments, where lipopolysaccharide- (LPS-) treated HepG2 and Hep3B cells were incubated with GW1929 or GW9662 to upregulate or downregulate the expression of PPAR*γ*. We found that upregulated PPAR*γ* ameliorated LDH release and improved cell viability. Our results indicated that increased expression of PPAR*γ* reduced ROS levels and inhibited the TXNIP/NLRP3 signaling pathway, resulting in decreased pyroptosis and reduced liver dysfunction during sepsis.

## 1. Introduction

Sepsis is a life-threatening organ dysfunction caused by a dysregulated host response to infection and is a major global public health challenge [1]. As one of the most vulnerable organs, the liver can be damaged at any stage during sepsis. Severe liver dysfunction often results in a poor prognosis during sepsis [2]. The mechanism of sepsis-induced liver injury is complex and involves many signaling pathways. Overproduction of reactive oxygen species (ROS), induced by mitochondrial dysfunction, is thought to play an important role in the pathogenesis of many different diseases, such as organ dysfunction in sepsis [3–5]. ROS can not only directly damage cells or tissues but also indirectly activate a series of damage-related signaling pathways [6, 7]. Recent studies have revealed that ROS is a fundamental factor in triggering pyroptosis by activating the NOD-like receptor 3 (NLRP3) inflammasome [8, 9].

Pyroptosis is a newly discovered programmed cell death process that occurs in many organs, including the liver, during acute or chronic inflammation. Pyroptosis is induced by inflammasomes, leading to the release of cellular contents, particularly the inflammatory mediators IL-1*β* and IL-18, eventually causing excessive inflammatory responses [10, 11]. NLRP3 proteolyzes pro-caspase-1 into cleaved caspase-1, which converts pro-IL1*β* into IL-1*β*. This process exerts a protective effect during the early stages of sepsis [12, 13]. A constant increase in the NLRP3-mediated release of these proinflammatory cytokines into tissues induces an excessive inflammatory response and leads to tissue damage. Recent studies have found that NLRP3-mediated pyroptosis leads to the depletion of immune cells, thus aggravating organ dysfunction during sepsis [14, 15]. However, few studies have revealed the importance of pyroptosis in sepsis-induced liver injury. Nevertheless, inhibition of pyroptosis may be a novel therapeutic approach for sepsis-induced liver injury.

Peroxisome proliferator-activated receptor gamma (PPAR*γ*) is an important member of the PPAR family. Its biological functions are complex and diverse. PPAR*γ* plays a significant role in protecting the liver from inflammation, oxidation, fibrosis, accumulating fat, and tumors. However, studies on the role of PPAR in sepsis-induced liver injury are still scarce. PPAR*γ* is a nuclear receptor that negatively regulates inflammation induced by ROS under either infectious or pathological conditions. Chorley et al. [16] found that activation of PPAR*γ* directly regulates the Nrf2 pathway, which mediates the expression of antioxidant defense genes. ROS is considered a crucial factor in the development of pyroptosis as it increases the expression of thioredoxin-interacting protein (TXNIP); PPAR*γ* thus could be a potential target for pyroptosis [17]. We hypothesized that PPAR*γ* inhibits overproduction of ROS by promoting Nrf2 expression to reduce NLRP3-mediated pyroptosis and alleviates sepsis-induced liver injury. This study is aimed at determining whether PPAR*γ* affects septic liver injury and at elucidating the underlying potential mechanisms.

## 2. Materials and Methods

### 2.1. Animals

Six-week-old C57BL/6 male mice were obtained from the Animal Feeding Center of Xi'an Jiaotong University Health Science Center. All animals were maintained in a specific pathogen-free room with constant temperature (23°C) and 12 h light and night exposure. All laboratory procedures were authorized by the Institutional Animal Care and Use Committee of the Ethics Committee of Xi'an Jiaotong University Health Science Center, China.

### 2.2. Sepsis Model

To create a sepsis model, mice were anesthetized with sodium pentobarbital (50 mg/kg, ip, once) and received a 1.5 cm midline laparotomy to expose the cecum. The cecum was lighted almost 1/3 tip and punctured twice with a 14-gauge needle, and the cecal contents were squeezed from the perforation site and placed back. Then, the abdominal cavity was closed in layers. All animals were returned to their cages keeping the body temperature at 36-38°C until completely recovery. In the control group, the mice were only incised in the abdomen without perforating and ligating the cecum [18].

### 2.3. Cell Culture and Cell Treatment

HepG2 and Hep3B cells were purchased from the Cell Bank of Shanghai Institutes for Biological Science (Shanghai, China). HepG2 and Hep3B cells were grown in DMEM medium at 37°C with 5% CO_2_. DMEM medium was supplemented with 10% fetal bovine serum (FBS), 100 U/mL penicillin, and 100 mg/mL streptomycin. HepG2 and Hep3B cells (1 × 10^5^ cells mL^−1^) were plated in 24-well plates in DMEM medium. Washing twice with PBS, the cells were stimulated with 1 *μ*g/mL LPS to create an activated inflammatory cell model. Twenty-four hours after stimulation, cell supernatants and HepG2 and Hep3B cells were collected to biochemical analysis.

### 2.4. Experimental Design

Mice were divided into four groups: control, cecal ligation and puncture (CLP), CLP+GW9662 (GW96), and CLP+GW1929 (GW19) (*n* = 20 for each group). Mice in the control group underwent a sham laparotomy. Mice in the CLP group underwent CLP. To explore the protective effect of PPAR*γ* on septic mice, mice in the CLP+GW96 and CLP+GW19 groups were intraperitoneally injected with GW9662 (PPAR*γ* inhibitor, 10 mg/kg) or GW1929 (PPAR*γ* agonist, 20 mg/kg) one hour before CLP operation. The liver tissues and blood samples were harvested from five anesthetized mice. The other 15 mice were fed for 120 h to determine the survival rate and body weight changes. The CLP+GW19+CDDO group (positive-control group) was intraperitoneally injected with GW1929 (10 mg/kg) and CDDO-EA (40 mg/kg).

To explore the role of PPAR*γ* in sepsis, HepG2 and Hep3B cells were pretreated for 30 min with GW9662 (5 *μ*M) or GW1929 (10 *μ*M) to downregulate or upregulate the expression of PPAR*γ*, respectively, and then stimulated with LPS to mimic inflammatory stimulation. The positive control group was treated with GW1929 (10 *μ*M) and CDDO-EA (20 *μ*M) to upregulate the expression of both PPAR*γ* and Nrf2.

### 2.5. Histologic Analysis

Twenty-four hours after CLP, a proportion of mouse liver specimens was harvested and fixed with formalin for paraffin embedding. Hematoxylin and eosin (HE) staining was performed according to the standard protocol. Histological score in the liver was evaluated by quantitative measurement of tissue damage through a blinded observer way with a light microscope. Liver histological score was the sum of the individual score grades from 0, minimal damage; 1, mild damage; 2, moderate damage; to 3, severe damage for each of the following 6 items: cytoplasmic color fading, vacuolization, nuclear condensation, nuclear fragmentation, nuclear fading, and erythrocyte stasis, ranging from 0 to 18 [19]. A representative field was chosen for application.

### 2.6. Quantification of Organ Function and Injury

Twenty-four hours after CLP, the mice were sacrificed to obtain the serum samples for evaluating the function of the liver. The liver function was estimated by testing the levels of serum total bilirubin (TBIL), aspartate aminotransferase (AST), and alanine aminotransferase (ALT). The levels of AST, ALT, and TBIL were measured by an automated procedure in the First Affiliated Hospital of Xi'an Jiaotong University.

### 2.7. Cytokine Levels

The liver tissue and cell supernatant were used to measure the release of IL-1*β*, IL-6, and TNF-*α* via commercial kits (San Ying Biotechnology, China) according to the manufacturer's instructions.

### 2.8. Dihydroethidium (DHE) Staining and ROS Activity Assay

Twenty-four hours after the LPS/CLP, HepG2 cells and liver tissues were immediately processed through DHE staining, as previously described [20]. The sections were photographed by a fluorescence microscope and the fluorescence intensity calculated with ImageJ software. The liver tissues were also collected to measure the levels of malondialdehyde (MDA), glutathione (GSH), and superoxide dismutase (SOD) by commercial biochemical kits (Nanjing Jiancheng, China).

### 2.9. Immunofluorescence (IF) Staining

Immunofluorescence staining for PPAR*γ*, Nrf2, and TXNIP expression was performed as previously described [21]. In short, the cells and tissue sections were washed with PBST and blocked with 5% goat serum and incubated with the primary antibody rabbit anti-PPAR*γ* (1 : 200, CST, USA), Nrf2 (1 : 200, CST, USA), and TXNIP (1 : 200, San Ying Biotechnology, China) overnight at 4°C. After washing again, the cells and tissue sections were incubated with the fluorescent secondary antibody (goat anti-rat antibody or goat anti-rabbit antibody, Servicebio, China, diluted 1 : 100) for 1 hour. Finally, the cells and tissue sections were counterstained with DAPI (4′-6-diamidino-2-phenylindole) and observed with a fluorescence microscope.

### 2.10. Western Blot Analysis

Briefly, protein in liver tissues was extracted using RIPA lysis buffer at 14,000 rpm for 15 min at 4°C. Protein concentration was determined using bicinchoninic acid (BCA). Protein was loaded on sodium dodecyl sulfate-polyacrylamide gel electrophoresis (SDS-PAGE) and transferred onto polyvinylidene difluoride (PVDF) membranes and blocked with 8% skim milk. Then, membranes were incubated with 1 : 1000 dilution of rabbit anti-Nrf2 antibody (CST, USA), anti-HO-1 antibody (San Ying Biotechnology, China), anti-ASC antibody (CST, USA), anti-TXNIP antibody (San Ying Biotechnology, China), anti-NLRP3 antibody (Beyotime Biotechnology, China), anti-ASC antibody (San Ying Biotechnology, China), anti-caspase-1 antibody (Abcam, USA), and anti-IL-1*β* antibody (Abcam, USA) and 1 : 10,000 dilution of mouse anti-*β*-actin antibody (San Ying Biotechnology, China) overnight at 4°C. After washing with PBST, the blots were incubated with 1 : 10,000 dilution of secondary antibodies (Abmart, China). Finally, the proteins were observed and photographed with the ECL (electrochemiluminescence) system. The protein electrophoresis bands were analyzed by the ImageJ software normalized to *β*-actin as a reference.

### 2.11. Immunohistochemical (IHC) Analysis

A proportion of liver tissue specimens was harvested and fixed with formalin for paraffin-embedded and cut into sections 4 *μ*m thick. Immunohistochemistry was used to measure the expression of NLRP3 and 8-OHdG. Briefly, each section was incubated with primary antibodies against 8-OHdG (1 : 200; San Ying Biotechnology, China) and NLRP3 (1 : 200; Beyotime Biotechnology, China) overnight at 4°C after a series of procedures (deparaffin, antigen retrieval, rinse, and block). The samples were washed 3 times with PBS and incubated with secondary antibodies at 37°C for 30 min and then and enriched with DAB (diaminobenzidine tetrahydrochloride) and counterstained with hematoxylin and observed with microscopic examination.

### 2.12. Cell Viability Assay and LDH Detection

The cell viability was evaluated with the Cell Counting Kit-8 (CCK-8, Abcam, US) following the manufacturer's description. The optical density (OD) values were measured at 450 nm.

Twenty-four hours after LPS stimulation, the LDH activity was measured by commercial kit (Nanjing Jiancheng, China). Mixing the 100 *μ*L reaction and 100 *μ*L supernatant in 96-well plates for 0.5 hours at 37°C, the absorbance of samples was tested by a microplate reader at 490 nm.

### 2.13. Statistical Analysis

The measurement data were shown as mean ± SD, and categorical data were shown as the number (percentage) in the group. Differences among multiple groups were assessed by one-way analysis of variance followed by the Student-Newman-Keuls post hoc test to determine significant differences. Kaplan-Meier survival curves and log-rank test were used to estimate the survival rate among multiple groups. To test whether the activation of PPAR*γ* alleviated weight changes in septic mice, repeated measures MANOVA was run on 5 days for the three groups, using Bonferroni post hoc comparisons to determine significant differences. All tests were two-tailed, and *P* value of <0.05 was considered to indicate statistical significance. The GraphPad Prism software (USA) was used to make the figure.

## 3. Results

### 3.1. PPAR*γ* Alleviated Sepsis-Induced Liver Injury

**Figure 1:**
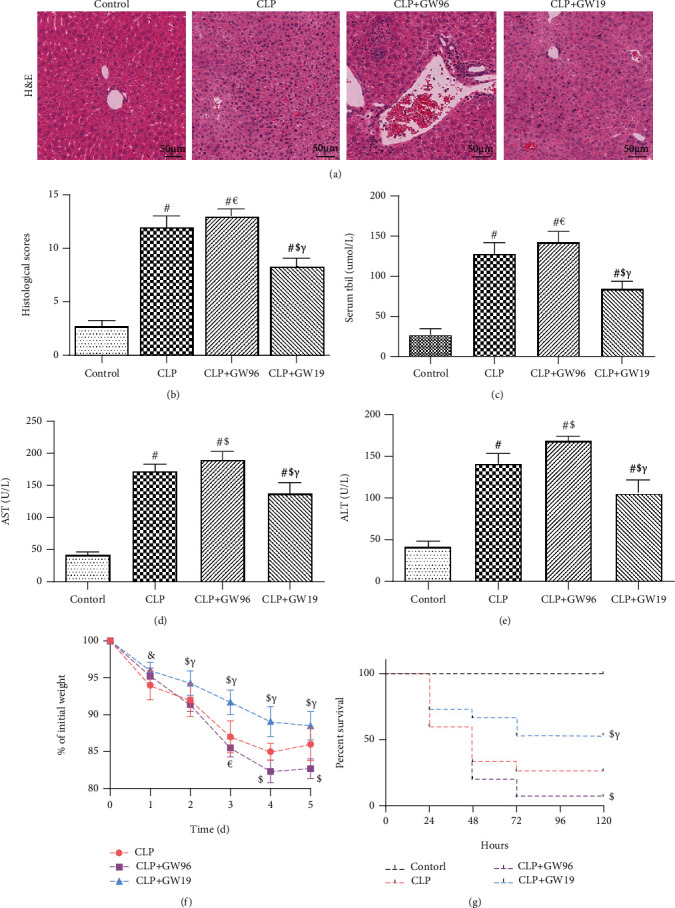
PPAR*γ* reduces sepsis-induced liver injury. Liver tissue and blood samples were collected 24 h after CLP from 5 mice and subjected to HE staining and liver function analysis. Fifteen mice were fed for 120 h to calculate the survival rate and body weight changes. (a) Hematoxylin and eosin staining (H&E) of representative liver sections (scale bars: 50 *μ*m). (b) Histological score. The levels of (c) TBIL, (d) AST, and (e) ALT. (f) The weight changes of mice 5 days after CLP. (g) Kaplan-Meier survival curve for mice 5 days after the CLP operation. (Data are shown as the mean ± SD. ^∗^*P* < 0.05 versus the control group; ^#^*P* < 0.01 versus the control group; ^€^*P* < 0.05 versus the CLP group; ^$^*P* < 0.01 versus the CLP group; ^&^*P* < 0.05 versus the CLP+GW96 group; *^γ^P* < 0.01 versus the CLP+GW96 group).

To explore the effect of PPAR*γ* on septic liver damage, blood samples and liver tissue were collected for liver function determination and histological evaluation. Mice were intraperitoneally administered GW9662 (10 mg/kg) or GW1929 (20 mg/kg) to downregulate or upregulate PPAR*γ* expression one hour before CLP operation. Hematoxylin and eosin (HE) staining revealed CLP induced marked congestion, inflammatory cell infiltration, necrosis, and degeneration in the liver. It is noteworthy that upregulation of PPAR*γ* by GW1929 attenuated the pathological changes in the liver. The histological scores also showed notable damage in septic mice 24 h after CLP. Upregulated PPAR*γ* in the CLP+GW19 group exhibited reduced histological damage compared to that in the CLP and CLP+GW96 groups (*P* < 0.0001) (Figures 1(a) and 1(b)). We next determined liver function by measuring the levels of total bilirubin (TBIL), aspartate aminotransferase (AST), and alanine aminotransferase (ALT). On comparing the levels between control and CLP groups, the results showed that the levels of TBIL, AST, and ALT were significantly increased after CLP (*P* < 0.0001). The CLP+GW96 group showed the highest increase among the groups. The upregulation of PPAR*γ* attenuated this increasing trend (Figures 1(c)–1(e)). Moreover, the mice were monitored to evaluate their weight changes and 5-day survival rates (Figures 1(f) and 1(g)). The results showed that the weight of mice in the CLP+GW19 group was lower than that of mice in the CLP and CLP+GW96 groups (*P* < 0.05). In addition, log-rank test analysis of the 5-day survival curves for CLP-induced sepsis demonstrated that the survival rate of the CLP+GW19 group was higher than that of the CLP and CLP+GW96 groups (*P* = 0.0112). Based on these results, we conclude that PPAR*γ* has a protective effect on liver function during sepsis.

### 3.2. PPAR*γ* Reduced ROS Injury in Sepsis-Induced Liver Injury

**Figure 2:**
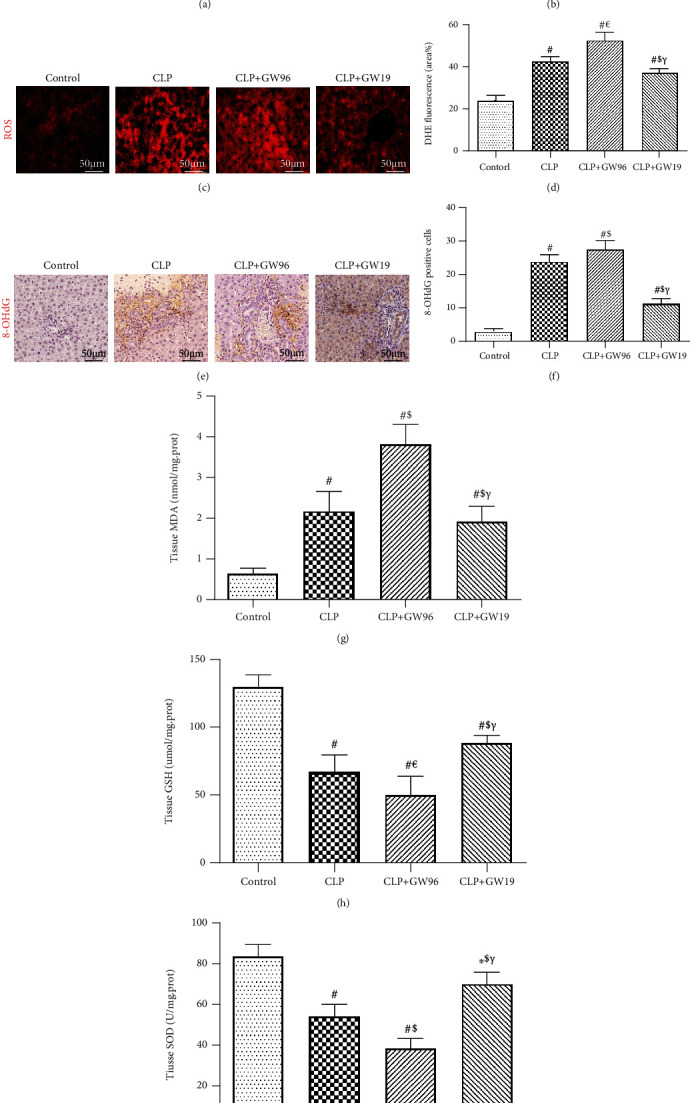
PPAR*γ* reduces ROS injury in sepsis-induced liver injury. (a, b) Dihydroethidium (DHE) fluorescence staining and fluorescence intensity of HepG2 cells (scale bars: 50 *μ*m). (c, d) DHE fluorescence staining and fluorescence intensity of the liver (scale bars: 50 *μ*m). (e, f) 8-OHdG immunohistochemical staining and the rate of positive cells in the liver (scale bars: 50 *μ*m). The levels of (g) malondialdehyde (MDA), (h) glutathione (GSH), and (i) superoxide dismutase (SOD). (*n* = 5; data are shown as the mean ± SD. ^∗^*P* < 0.05 versus the control group; ^#^*P* < 0.01 versus the control group; ^€^*P* < 0.05 versus the CLP group; ^$^*P* < 0.01 versus the CLP group; ^&^*P* < 0.05 versus the CLP+GW96 group; *^γ^P* < 0.01 versus the CLP+GW96 group).

PPAR*γ* negatively regulates ROS during inflammation. ROS are crucial components of pyroptosis. To clarify the inhibitory effect of PPAR*γ* on pyroptosis, we used DHE staining to measure ROS levels in both HepG2 cells and liver tissues after LPS or CLP. The DHE fluorescence intensity was significantly increased after LPS or CLP, especially in the CLP+GW96 group. Upregulated PPAR*γ* significantly attenuated this effect (Figures 2(a)–2(d)). 8-OHdG is a product of oxidative DNA damage caused by ROS. To further determine the antioxidative property of PPAR*γ*, we used immunohistochemically stained 8-OHdG (Figures 2(e) and 2(f)). The results of IHC staining showed that in comparison to the CLP and CLP+GW96 groups, upregulation of PPAR*γ* in the CLP+GW19 group decreased the number of 8-OHdG-positive cell. Moreover, the levels of malondialdehyde (MDA) in the CLP group were increased compared with those in the control group. The CLP+GW96 group showed a greater increase in MDA levels than the CLP+GW19 group. The levels of glutathione (GSH) and superoxide dismutase (SOD) considerably decreased in the CLP+GW96 group, and this decrease was reversed by the activation of PPAR*γ* (Figures 2(g) and 2(i)). These results confirmed that PPAR*γ* reduced sepsis-induced ROS injury in the mouse liver.

### 3.3. PPAR*γ* Inhibited ROS Injury via Activation of Nrf2

**Figure 3:**
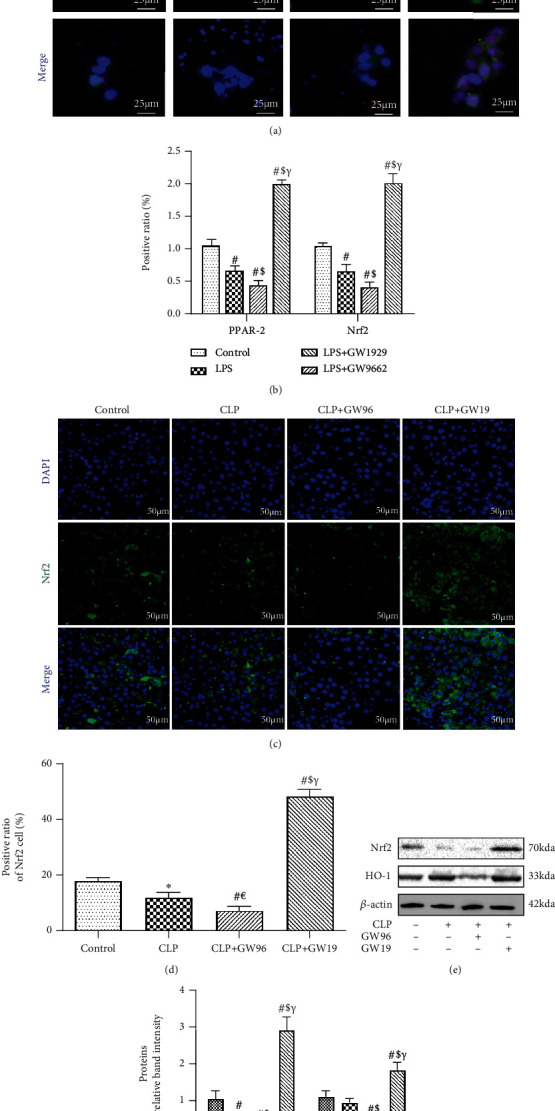
PPAR*γ* inhibits ROS injury via the activation of Nrf2. (a, b) Immunofluorescence staining for PPAR*γ* and Nrf2 and the rate of positive HepG2 cells (scale bars: 25 *μ*m). (c, d) Immunofluorescence staining for Nrf2 and the rate of positive cells in the liver (scale bars: 50 *μ*m). (e, f) Representative immunoblots to detect Nrf2 and HO-1 following protein extraction from the liver 24 h after CLP. (*n* = 5; data are shown as the mean ± SD. ^∗^*P* < 0.05 versus the control group; ^#^*P* < 0.01 versus the control group; ^€^*P* < 0.05 versus the CLP group; ^$^*P* < 0.01 versus the CLP group; ^&^*P* < 0.05 versus the CLP+GW96 group; *^γ^P* < 0.01 versus the CLP+GW96 group).

Nrf2 is the master regulator of ROS balance. To further explore the protective effect of PPAR*γ* on ROS injury, we used IF staining to measure the expression of Nrf2 under conditions of differential PPAR*γ* expression (Figures 3(a) and 3(b)). Nrf2 expression decreased in HepG2 cells 24 h after LPS stimulation. GW96 treatment significantly inhibited the activation of Nrf2 compared to the LPS group. The number of Nrf2 positive cells was higher in the LPS+GW19 group than in the LPS and LPS+GW96 groups. In addition, we used IF staining to measure the expression of Nrf2 in the liver tissues (Figures 3(c) and 3(d)). These results above were consistent with those of in vitro experiments, indicating that upregulated PPAR*γ* promoted the expression of Nrf2 to reduce ROS injury. Western blot analysis was used to measure the effect of PPAR*γ* on Nrf2 and its downstream protein HO-1 (an antioxidant stress damage protein). The expression of Nrf2 and HO-1 was reduced significantly in the CLP and CLP+GW96 groups compared to that in control group, and this effect was reversed by GW1929 treatment, consistent with the results of IF staining (Figures 3(e) and 3(f)). Based on these results, we concluded that PPAR*γ* inhibited ROS injury via activation of the Nrf2 signaling pathway.

### 3.4. PPAR*γ* Suppressed Inflammatory Responses and Pyroptosis in Septic Liver Injury

**Figure 4:**
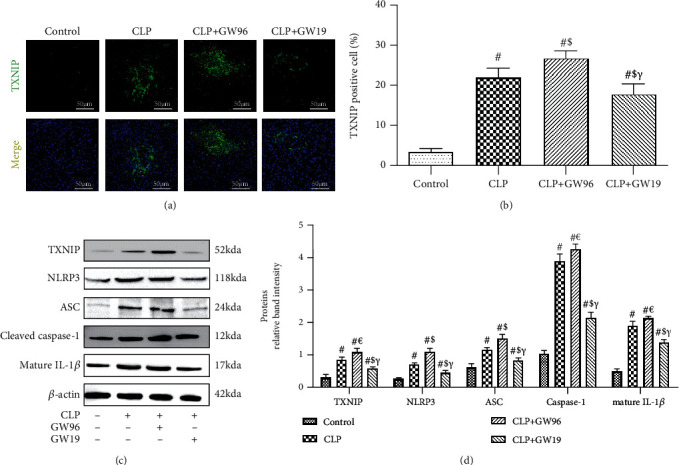
PPAR*γ* inhibits the TXNIP/NLRP3 signaling pathway. (a, b) IF staining for TXNIP and the rate of positive cells in the liver (scale bars: 50 *μ*m). (c) Representative western blot and (d) quantitative analysis of TXNIP, NLRP3, ASC, cleaved caspase-1, and mature IL-1*β* levels normalized against *β*-actin 24 h after CLP. (*n* = 5; data are shown as the mean ± SD. ^∗^*P* < 0.05 versus the control group; ^#^*P* < 0.01 versus the control group; ^€^*P* < 0.05 versus the CLP group; ^$^*P* < 0.01 versus the CLP group; ^&^*P* < 0.05 versus the CLP+GW96 group; *^γ^P* < 0.01 versus the CLP+GW96 group).

We next determined the expression of TXNIP in the livers of septic mice (Figures 4(a) and 4(b)). TXNIP expression was elevated during septic liver injury but decreased after the upregulation of PPAR*γ*. We then used western blot analysis to measure the expression of members of the TXNIP/NLRP3 signaling pathway in vivo. The results showed that the expression of TXNIP, NLRP3, cleaved caspase-1, apoptosis-associated speck-like protein (ASC), and mature IL-1*β* was significantly increased whereas the expression of Nrf2 was significantly reduced in the CLP+GW96 and CLP groups compared with that in the control group. GW1929 treatment effectively promoted the expression of Nrf2 and inhibited the activation of the TXNIP/NLRP3 signaling pathway (Figures 4(c) and 4(d)). These results confirmed that PPAR*γ* activates Nrf2 to mitigate ROS damage, thereby inhibiting the expression of the TXNIP/NLRP3 signaling pathway to reduce sepsis-induced hepatocyte pyroptosis.

**Figure 5:**
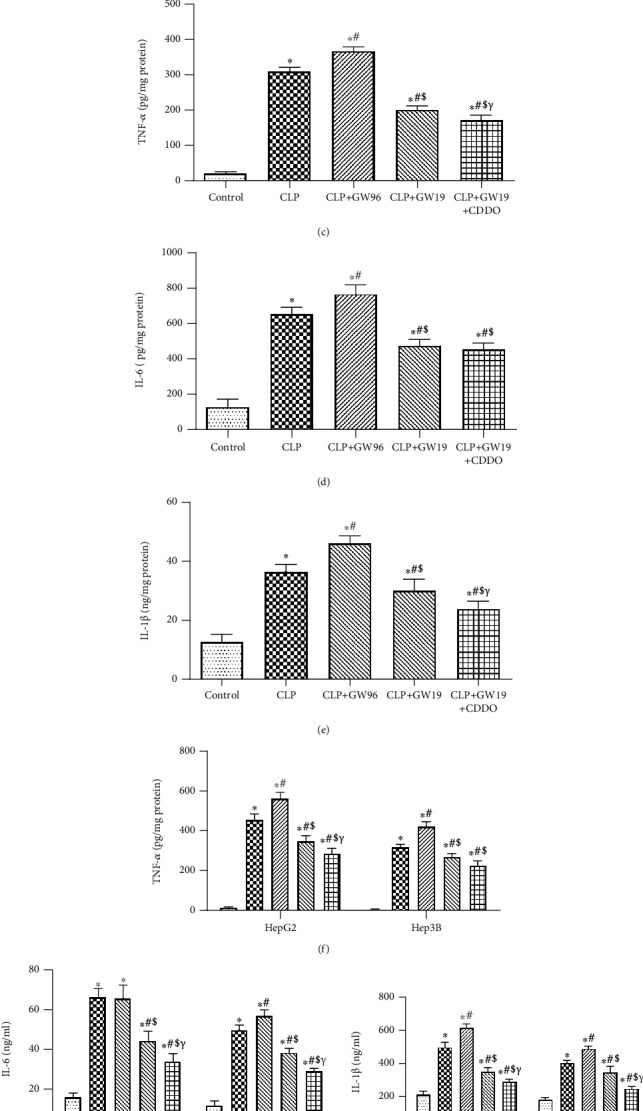
PPAR*γ* reduces sepsis-induced liver injury by alleviating inflammatory responses and pyroptosis. (a, b) NLRP3 immunohistochemical staining (scale bars: 50 *μ*m) and the rate of positive cells in the liver. (c–e) Levels of TNF-*α*, IL-6, and IL-1*β* in liver tissue. (f–h) The levels of TNF-*α*, IL-6, and IL-1*β* in HepG2 and Hep3B cell supernatants (*n* = 5). (i) LDH levels. (j) Cell viability. (*n* = 5; data are shown as the mean ± SD. ^∗^*P* < 0.05 versus the control group; ^#^*P* < 0.01 versus the control group; ^€^*P* < 0.05 versus the CLP group; ^$^*P* < 0.01 versus the CLP group; ^&^*P* < 0.05 versus the CLP+GW96 group; *^γ^P* < 0.01 versus the CLP+GW96 group).

To confirm that the protective effect of PPAR*γ* is related to NLRP3-medited pyroptosis in septic liver injury, we used IHC staining to determine the activity of the NLRP3 inflammasome (Figures 5(a) and 5(b)). Twenty-four hours after CLP, the expression of NLRP3 was increased compared to that in the control group. The PPAR*γ* inhibitor group showed the highest increasing trend among the three groups. Upregulated PPAR*γ* decreased the expression of NLRP3, whereas upregulation of both PPAR*γ* and Nrf2 showed the most obvious effects of inhibition among the groups. Calculation of the number of NLRP3-positive cells confirmed these results. We examined the levels of inflammatory factors in the liver tissue (Figures 5(c)–5(e)). The results showed that the levels of TNF-*α*, IL-6, and IL-1*β* were increased 24 h after CLP compared with those in the control group. The CLP+GW96 group showed the highest increase among the CLP groups. Activation of PPAR*γ* suppressed the inflammatory responses in the liver during sepsis. Activation of both PPAR*γ* and Nrf2 showed the most obvious effects among the groups. We also examined the levels of the three inflammatory factors in HepG2 and Hep3B cell supernatants (Figures 5(f)–5(h)), and the results were consistent with those of the in vivo experiments. We then measured LDH release and cell viability in HepG2 and Hep3B cells (Figures 5(i) and 5(j)). The results showed that PPAR*γ* could ameliorate LDH release and improve cell viability compared to the LPS and LPS+GW96 groups. Activation of both PPAR*γ* and Nrf2 showed the most obvious effects among the groups. Based on these results, we conclude that PPAR*γ* and Nrf2 activation can alleviate inflammatory responses and pyroptosis in mouse livers during sepsis.

## 4. Discussion

Sepsis is an intractable disorder characterized by multiorgan damage, oxidative stress, inflammatory cytokine stimulation, and altered circulation, but its pathogenesis remains largely unclear [22]. Studies have highlighted the important role of NLRP3-mediated pyroptosis in sepsis [23]. Reducing sepsis-induced damage by inhibiting pyroptosis is a potential therapeutic strategy. During sepsis, the liver is a vulnerable organ, as manifested by cholestasis, and can be damaged at any stage of sepsis. Septic liver injury often indicates poor prognosis [24]. Owing to the limited treatment options available for sepsis, reducing mortality resulting from sepsis is challenging in clinical practice.

PPAR*γ* is a ligand-activated transcription factor. Activation of PPAR*γ* modulates the expression of several genes involved in immunity, inflammation, metabolism, cell proliferation, and cell differentiation [25]. A study showed that PPAR*γ* agonists reduced the inflammatory response and oxidative stress in a model of nonalcoholic cirrhotic mice, thereby reducing liver damage [26]. This study demonstrated a protective effect of PPAR*γ* against septic liver injury. After CLP, HE staining showed marked congestion, inflammatory cell infiltration, necrosis, and degeneration in the liver tissues. Activation of PPAR*γ* effectively alleviated liver damage, improved liver function, and decreased the death rate in septic mice.

ROS play a key role in sepsis-induced injury. Mitochondrial damage induced by excessive ROS is a core event in sepsis-induced liver dysfunction. Studies have shown that PPAR*γ* protects against hepatic ischemia/reperfusion injury in mice by inhibiting action of ROS [27]. PPAR*γ* also protects against burn-induced oxidative injury [28]. In this study, we observed that PPAR*γ* strongly reduced ROS-mediated injury in hepatocytes during sepsis both in vivo and in vitro. DHE staining showed an increase in fluorescence intensity after LPS/CLP, particularly in the PPAR*γ* inhibitor group. MDA is an indicator of the lipid damage induced by ROS. After CLP, the secretion of MDA was markedly increased in the PPAR*γ* inhibitor group compared to the other CLP groups. Furthermore, SOD and GSH are critical enzymes involved in the defense against ROS. The secretion of SOD and GSH was significantly decreased in the PPAR*γ* inhibitor group compared to the CLP group. Results of IHC staining for 8-OHdG were consistent with these results. Our data suggest that PPAR*γ* protects against septic liver damage by reducing ROS-induced injury.

Nrf2 is an intracellular transcription factor that regulates the expression of a number of genes that encode antioxidant enzymes to reduce ROS-induced injury. In the acute injury phase, PPAR*γ* directly restricts tissue damage by stimulating the Nrf2/ARE axis to neutralize oxidative stress. After evaluating the expression of Nrf2 and its downstream molecule HO-1, we found that their expression was suppressed after CLP, especially following treatment with GW9662. GW1929 treatment significantly reversed this trend, indicating that PPAR*γ* inhibited ROS injury via activation of the Nrf2 pathway. The effect was most obvious when both PPAR*γ* and Nrf2 were activated.

High levels of ROS induce NLRP3 expression via TXNIP activation, indicating that TXNIP acts as a bridge between ROS and NLRP3-mediated pyroptosis [8]. In lupus nephritis, activation of Nrf2 inhibits gene transcription of the NLRP3 inflammasome and inhibits the production of IL-1*β*, a downstream product. Excessive ROS induces TXNIP activation. Activated TXNIP directly activates NLRP3 inflammasome and induces pyroptosis. Accumulating evidence indicates that NLRP3-mediated pyroptosis results in plasma membrane rupture and subsequently in a drastic release of powerful proinflammatory factors such as IL-1*β*, thereby resulting in a hyperinflammatory response to sepsis [29]. Therefore, we measured the expression of proteins of the TXNIP/NLRP3 signaling pathway. The data showed a marked upregulation in the expression levels of TXNIP, NLRP3, caspase-1, and IL-1*β*, suggesting that the NLRP3 inflammasome may contribute to liver dysfunction during sepsis. Notably, activation of the TXNIP/NLRP3 signaling pathway was markedly attenuated by GW1929 treatment. The levels of IL-1*β*, IL-6, and TNF-*α* were decreased by treatment with GW1929 indicating that the upregulation of PPAR*γ* inhibited the NLRP3-mediated inflammatory responses. In addition, IL-1*β*, IL-6, and TNF-*α* levels were significantly decreased following activation of both PPAR*γ* and Nrf2. Based on these results, we conclude that PPAR*γ* protects against septic liver damage by activating Nrf2 and decreases NLRP3-mdiated pyroptosis by inhibiting ROS-mediated injury.

The mechanism of septic liver damage is complex and involves multiple molecular pathways. This study revealed one possible mechanism of septic liver damage. However, these findings require further elaborate research and verification.

## 5. Conclusion

In conclusion, the current findings demonstrated that the activation of PPAR*γ* reduced ROS levels and inhibited the TXNIP/NLRP3 signaling pathway to decrease pyroptosis and reduce liver damage during sepsis. These findings may contribute to the development of novel therapeutic agents for attenuating sepsis-induced liver damage.

## Data Availability

The data related to mouse data and cell data used to support the findings of this study are available from the corresponding author upon request.

## Abbreviations

PPAR*γ*: Peroxisome proliferator-activated receptor
Nrf2: Nuclear factor erythroid 2-related factor 2
NLRP3: NOD-like receptor 3
TXNIP: Thioredoxin-interacting protein
MDA: Malondialdehyde
SOD: Superoxide dismutase
GSH: Glutathione
TNF-*α*: Tumor necrosis factor *α*
IL-6: Interleukin 6
IL-1*β*: Interleukin 1*β*
ROS: Reactive oxygen species
DHE: Dihydroethidium
CLP: Cecal ligation and puncture.
